# Inositols, Probiotics, and Gestational Diabetes: Clinical and Epigenetic Aspects

**DOI:** 10.3390/nu14081543

**Published:** 2022-04-08

**Authors:** Ester Vitacolonna, Maria Masulli, Luisa Palmisano, Liborio Stuppia, Marica Franzago

**Affiliations:** 1 Department of Medicine and Aging, School of Medicine and Health Sciences, “G. d’Annunzio” University, Chieti-Pescara, Via dei Vestini, 66100 Chieti, Italy; marica.franzago@unich.it; 2 Center for Advanced Studies and Technology (CAST), “G. d’Annunzio” University, Chieti-Pescara, 66100 Chieti, Italy; stuppia@unich.it; 3 Department of Clinical Medicine and Surgery, Federico II University, 80131 Naples, Italy; magia77@inwind.it (M.M.); lupalmi23@gmail.com (L.P.); 4 Department of Psychological, Health and Territorial Sciences, School of Medicine and Health Sciences, “G. d’Annunzio” University, Chieti-Pescara, 66100 Chieti, Italy

**Keywords:** gestational diabetes, nutrition, epigenetic regulation, fetal programming, Myo-inositol, D-chiro inositol, probiotics

## Abstract

There is growing interest in the potential role of different stereoisomers of inositol or their combination as well as probiotics supplementation in healthy glucose metabolism during pregnancy and in promoting offspring health. The aim of this review is to clarify the effects of several inositol and probiotics-based supplements in the prevention and treatment of gestational diabetes (GDM). Moreover, we will discuss the epigenetic aspects and their short- and long-term effects in response to probiotic intervention as well as the possible implications of these findings in guiding appropriate supplementation regimens in pregnancy.

## 1. Introduction

Nutritional interventions in pregnancy are based on dietary counseling and/or nutrient supplementation. Nutraceuticals are nutrients and/or bioactive compounds generally contained in some foods, especially of vegetable or microbial origin, and which have beneficial effects on human health [1]. They are bioactive ingredients for which the ability to strengthen health has been demonstrated in dosages that are higher than those which can be obtained from food. Nutraceuticals can be used as fortified foods, added to foods and drinks, or in the form of oral supplements.

Pregnancy is characterized by a physiological increase in insulin resistance which, in predisposed women, could induce alterations in glucose metabolism and gestational diabetes mellitus (GDM). It is known that some nutritional supplements in pregnancy can improve insulin resistance [2,3,4]. For this reason, there is growing interest from the scientific community in the role that supplementation can play in the prevention and treatment of hyperglycemia in pregnancy; particular attention has been paid to inositol and probiotics. GDM is defined as any degree of glucose intolerance that is first recognized during pregnancy and that is not clearly overt diabetes prior to gestation [5]. GDM is one of the most common diseases during pregnancy, with a global prevalence of 1% to 28%, depending on the diagnostic criteria used, and with considerable variability in different ethnic groups. Several strategies for screening and diagnosis are currently used, such as those of the American Diabetes Association (ADA) [5] and the World Health Organization (WHO) [6]. Following the HAPO Study [7], the International Association of the Diabetes and Pregnancy Study Groups (IADPSG) [8] recommended thresholds lower than those previously used when obtained through a 2 h 75 g oral glucose tolerance test (OGTT). Many GDM risk factors have been identified and widely described, including maternal obesity, weight gain during pregnancy, advanced maternal age, family history of diabetes, ethnicity, and sedentary lifestyle. In addition, it has been suggested that the interaction among genetics, epigenetics, and environmental factors may also play a role in GDM [9,10]. GDM, whose pathogenic mechanisms remain unclear, has been correlated with an increased risk of complications during pregnancy and with possible detrimental future health outcomes in both mother and offspring, including a high risk of cardiovascular disease, metabolic syndrome, and Type 2 diabetes mellitus (T2D). Notably, in terms of GDM prevention, it is a priority to promote healthy nutrition and lifestyle both in the peri-conceptional period and during pregnancy. Moreover, prevention strategies such as inositol and probiotics-based supplements have been proposed.

The aim of this review is to examine the effects of inositol- and probiotics-based supplements during pregnancy in the prevention and treatment of GDM. This could help physicians with an evidence-based approach to supplementation regimens in pregnancy and, in light of the recent advances in omics sciences, to the possible epigenetic effects of the various compounds.

A literature search was conducted using MEDLINE (https://www.ncbi.nlm.nih.gov/PubMed), EMBASE (https://www.embase.com/), SCOPUS (https://www.scopus.com/), Google Scholar (https://scholar.google.com), and the Cochrane database (https://www.cochranelibrary.com/), up to 16 March 2022, to obtain evidence-based data. Unpublished trials were searched in the www.clinicaltrials.gov register. The following keywords were used in the literature search: “oral probiotics supplementation”, “inositol” OR “Myo-inositol supplementation”, “D-chiro-inositol supplementation” “dietary supplementation” AND “pregnant women” OR “Gestational diabetes” OR “Gestational diabetes treatment” OR “Gestational diabetes prevention”.

## 2. Inositols

Inositol, a carbocyclic polyol, is synthesized by most vegetable and animal cells [11,12] and accumulates in the kidneys, brain, liver, placenta, and other tissues. Inositol mediates cell signal transduction and participates in many physiological processes, such as glucose and calcium metabolism, endocrine modulation, and the stress response [13,14]. Inositol exists in nine geometrical isomers by way of the epimerization of its hydroxyl groups. Myoinositol (MI) and D-chiro inositol (DCI) are the predominant forms under which it can be found in nature as well as in food and are its most clinically relevant forms. Both isomers can influence metabolism through different mechanisms; it has been also found that MI and DCI have insulin-like properties which are efficient in improving glycemic control, especially post-prandial blood glucose [15].

MI supplementation has been known to have therapeutic effects in infertile women [16] and seems to be useful during pregnancy in the prevention of some metabolic conditions. In this context, although other confirmatory studies are needed in order to understand the mechanisms involved, several studies have pointed out that MI administered early in pregnancy can prevent the onset of GDM, also suggesting a benefit to different categories of women at risk, including overweight and obese women, as well as patients affected by polycystic ovary syndrome (PCOS). In addition, it has been indicated that MI supplementation in pregnancy can be associated with protection from adverse maternal and fetal outcomes, such as hypertension, preterm birth, and large-for-gestational-age (LGA) babies [17].

Previous studies have shown that T2D is characterized by decreased chiro-inositol mediator bioactivity and chiro-inositol content [18]. Studies conducted on human adipocyte cell lines have shown that DCI plays a direct role in the differentiation and function of human adipocytes [19]. In addition, DCI has a role in the accumulation of lipids and on the number and size of lipid droplets in the later stages of adipocyte differentiation [19]. Further research is needed to better understand the role of DCI on fat metabolism in women with PCOS, obesity, and menopause.

An observational study by Scioscia et al. [20] showed that increased urinary excretion of inositol phosphoglycan in GDM-affected women and in patients with T2D was positively related to blood glucose levels. In addition, urinary excretion of MI and DCI in early pregnancy was also higher in women with GDM, when compared with women without GDM [21]. Recently, Pillai et al. [22] showed that placental inositol concentration was lower in GDM-affected women than in controls; also, higher maternal mid-gestation glycemia was associated with lower placental inositol. In addition, an increase in fasting glycemia has been associated with lower levels of the MI synthesis enzyme proteins and transporters, the expression of which was also correlated with placental inositol content. Therefore, this novel study hypothesized that glycemia-induced dysregulation of placental inositol synthesis and transport may be implicated in reduced placental inositol content in GDM, and this may lead to accelerated fetal growth [22].

### 2.1. Inositol Supplementation and Gestational Diabetes

#### 2.1.1. Inositol Supplementation and GDM Prevention

In early pregnancy (12–13 weeks of gestation), supplementation with MI 2000 mg + folic acid 200 mcg twice a day has been demonstrated to reduce the incidence of GDM in at-risk women. MI supplementation in pregnant women with a family history of T2D reduces GDM incidence and the delivery of macrosomic fetuses [23,24]. Matarrelli et al. [25] confirmed the protective effect of supplementation with MI 2000 mg + folic acid 200 mcg vs placebo on GDM incidence in mid-pregnancy (RR 0.127; *p* = 0.001). In addition, women treated with MI showed better maternal and fetal outcomes (less insulin therapy, delivery at a later gestational age, and less neonatal hypoglycemia and macrosomia). Moreover, MI supplementation in obese [26] or overweight [27] pregnant women, when introduced in the first trimester, significantly reduced the incidence of GDM (OR, 0.36; *p* < 0.001) and reduced insulin resistance evaluated by HOMA index [26]. Furthermore, a secondary analysis of databases from three RCTs [24,26,27] showed that supplementation with MI throughout pregnancy reduced the risk of preterm birth (OR, 0.44; *p* = 0.03) and macrosomia (OR, 0.38; *p* = 0.04) when compared with controls [28]. These results were confirmed by recent meta-analyses, showing a reduction in the GDM rate [29,30], preterm delivery [29], and lower glycemic values during OGTT in at-risk women [30]. Treatment with MI is associated with a 66% reduction in the risk of developing GDM (four studies, RR 0.34, 95% CI 0.20, 0.58), with a low number needed to treat (NNT 4.85, 95% CI 4.79, 4.91) [31].

With regard to the MI/DCI association, Farren et al. found that supplementation with a combined dose of 1100 mg MI, 27.6 mg DCI, and 400 μg folic acid did not reduce GDM incidence in women with a family history of diabetes (*p* = 0.34) [32]. It has been hypothesized [33] that the lack of preventive effect of inositol, shown by Farren et al., could be related to the dose of the supplement used, which is different than that (4 g MI) used in other trials [24,27].

A trial evaluating the effects of different inositol stereoisomers and dosages on the HOMA-IR index in the prevention of GDM in women affected by high fasting glycaemia in the first trimester of pregnancy [4] showed that the group randomized to MI alone had a lower incidence of GDM [4]. Two study reviews showed, not only the safety and tolerability of MI supplementation, but also that the daily dose of 4000 mg MI may be promising in reducing the risk of GDM and preterm birth rate [34], while also improving insulin sensitivity and glycemic homeostasis [35].

The results of the NiPPeR study [36] have reignited the debate on the role of MI in GDM prevention. NiPPeR is a double-blind RCT including 1729 women who were planning to conceive. The women enlisted were from the United Kingdom, Singapore, and New Zealand. The purpose was to investigate whether a nutritional formulation containing MI (4 g/day), probiotics, and multiple micronutrients (intervention), taken before conception and during pregnancy, could improve pregnancy outcomes compared to standard micronutrient supplementation. The results showed that there were no significant effects on the incidence of GDM (24.8% vs. 22.6%, adjusted risk ratio [aRR] 1.22 [0.92–1.62]) and macrosomia (adjusted β = 0.05 kg [−0.03 to 0.13]). However, the treatment reduced pre-term births (5.8% vs. 9.2%, aRR 0.43 [0.22–0.82]), confirming the results of previous studies on MI. A reduction in the incidence of major postpartum bleeding was also found.

Table 1 summarizes the general characteristics of the main RCTs on inositol supplementation and GDM prevention.

In summary, MI at a daily dose of 4000 mg can be considered in the prevention of GDM in at-risk women. Multicenter studies on larger populations are needed in order to assess the optimal dosage, frequency, and timing of supplementation, and the safety and long-term effects on maternal, neonatal, and childhood outcomes.

#### 2.1.2. Inositol Supplementation and GDM Treatment

Studies on the efficacy of inositols in the treatment of GDM are scarce and results are inconsistent. Corrado et al. showed that, in women with GDM, an 8-week supplementation with MI at a dosage of 2000 mg plus 200 mcg of folic acid twice per day reduced plasma adiponectin (*p* = 0.009) and HOMA-IR (*p* = 0.0001) when compared to supplementation with folic acid alone [37].

Recent literature has provided insight into the use of different stereoisomers of inositol or their combination in GDM treatment [2,38], showing an improvement in fetal growth, glycemic control (post-prandial glycemia and insulin doses), and maternal weight gain in women who received DCI 500 mg twice a day vs placebo [38]; remarkably, a significant reduction in HOMA-IR and a lower need for intensified insulin therapy in women exposed to MI and MI plus DCI (2) have also been demonstrated. In a pilot case-control study, Pintaudi et al. [3] found lower mean glucose levels and an improvement in glucose variability after MI treatment in women affected by GDM, suggesting that MI supplementation can be a useful strategy for treating GDM. The safety profile of MI, even at a high dosage during GDM, appears free of any side effects; in addition, the faster glucose-lowering effect due to a higher dose of MI may open new therapeutic perspectives in the treatment and prevention of GDM [39].

An RCT (2) comparing the effect of different dosages of inositol stereoisomers supplementation—400 mcg folic acid (control treatment), 4000 mg MI plus 400 mcg folic acid (MI treatment), 500 mg DCI plus 400 mcg folic acid (DCI treatment), or 1100/27.6 mg MI/DCI plus 400 mcg folic acid (MI plus DCI treatment)—on insulin resistance levels and several maternal-fetal outcomes in GDM women showed that inositol supplementation (MI and MI+DCI) improved insulin resistance (*p* < 0.001), reduced weight gain, and improved some pregnancy outcomes.

A more recent RCT, performed in 100 Asian women with GDM diagnosed between 14–28 weeks’ gestation, has shown that supplementation with MI in a dose of 1000 mg twice daily, when started soon after the diagnosis of GDM, is effective in achieving glycemic control and decreasing the need for additional pharmacological therapy [40].

Table 2 summarizes the general characteristics of main RCTs on inositol supplementation and GDM treatment.

Further clinical studies are warranted in order to define the optimal dose, frequency, and timing of MI supplementation, as well as to explore its possible adverse and long-term effects on women affected by GDM. In particular, low inositol concentrations have been observed in neural tube defects (NTDs) which are related to both maternal diabetes and obesity. The results obtained from inositol supplementation in women at a higher risk of offspring with NTDs seem promising. In this context, Gambioli et al. [16] suggested that MI supplementation, occurring at least one month before conception and until the 36th week of pregnancy, seems to reduce the risk of both NTD and GDM. Since no major side effects have been identified, MI can also be considered as a possible useful strategy in NTDs prevention in the preconception period [16].

## 3. Probiotics

Probiotics are live and viable micro-organisms. When administered at therapeutical dosages, they can have health benefits to the host by influencing their gut flora and/or modifying their immune system [41,42].

The main actions of probiotics, which are consumed in the form of yoghurt, fermented milks, fermented foods, or supplements, include enhancing mucosal barrier function, antagonizing pathogens, inhibiting bacterial adherence, invading the intestinal epithelium, boosting the immune system, and regulating the central nervous system [43].

Prebiotics are selectively fermented ingredients triggering specific changes in the composition and/or activity of the microbiota, thus being able to benefit the host [43]; symbiotics, on the other hand, consist of a mixture of prebiotics and probiotics which can improve the survival and implantation of live microbial supplements in the gastrointestinal tract, thus beneficially affecting the host.

Studies on the efficacy and safety of probiotics have shown several critical issues related to the non-homogeneity of study designs, small sample sizes, and the use of multiple single or combined strains.

### 3.1. Probiotics and Pregnancy

The microbiota is influenced by several factors: among others, the interaction between diet and environment, the host’s genetic makeup and immune system, and the microbial strains which make up the microbiota itself. Although its underlying mechanisms remain to be clarified, the gut microbiota (GM) undergoes profound alterations in pregnancy; between the first and third trimester, there is an overall increase in Proteobacteria and Actinobacteria, which are involved in the metabolism of nutrients, the strengthening of the intestinal barrier, and a decrease in short chain fatty acids (SCFAs) bacterial production [44]. Maternal metabolism disorders can also cause an imbalance in the microbiota; one form of alteration, known as dysbiosis, can increase the risk of pre-eclampsia, diabetes, infections, and pre-term birth. Furthermore, neonatal intestinal dysbiosis appears to play an important role in both pathological processes and long-term metabolic health [45].

The role of probiotic supplementation is currently discussed with controversial results, and to date, the debate on the preventive or therapeutic effects of probiotic supplements in pregnancy through the modulation of the GM is still ongoing. Probiotics are generally considered safe to pregnant women and their fetuses [46].

It has been suggested that supplementation including specific probiotics and symbiotics during pregnancy can improve insulin sensitivity [47,48], potentially by modulating the anti-inflammatory response [49] and the upregulation of genes related to fat metabolism and insulin sensitivity in the gut and epididymal fat tissue [48]. In light of this evidence, supplementation with probiotics could have a rationale for use to mitigate insulin resistance and reduce the risk of obesity and diabetes. However, data are contrasting, and further investigation is needed.

### 3.2. Probiotics and Gestational Diabetes

#### 3.2.1. Probiotics Supplementation and GDM Prevention

Winkins et al. [50] showed that probiotics supplementation from 14 to 16 weeks’ gestation can reduce GDM incidence, particularly among older women (RR 0.31; *p* = 0.009) and those with previous GDM (RR 0.00; *p* = 0.004). Previous studies have shown that, even in normoglycemic populations, probiotics can improve blood glucose control, insulin sensitivity, markers of insulin metabolism, triglycerides, biomarkers of inflammation, and oxidative stress [51,52,53]. However, these findings were not confirmed by other studies [54].

The SPRING Trial, conducted in overweight and obese pregnant women, showed that probiotics (Lactobacillus rhamnosus and Bifidobacterium animalis subspecies lactis), administered from the first half of the second trimester, do not prevent GDM at 28 weeks’ gestation [55]. Furthermore, the Healthy Mums and Babies (HUMBA) trial, conducted in a multiethnic, highly-deprived population of pregnant women with obesity, showed no benefits of probiotics (Lactobacillus rhamnosus GG and Bifidobacterium lactis BB12) on gestational weight gain, GDM, or birthweight [56]. A Cochrane review of GDM prevention examined several dietary supplements, such as probiotics, and concluded that no intervention resulted in clear benefit or harm [57]. Similarly, a systematic review and meta-analysis including 17 randomized controlled trials (RCTs) showed that probiotics during pregnancy do not reduce the incidence of GDM (MH-OR: 0.77 [0.51,1.16], *p* = 0.21, I2:62%), with a very small, statistically but not clinically significant, reduction in fasting plasma glucose (mean difference −1.01 [−1.96, −0.06] mg/dL, *p* = 0.02, I2:46%). Among secondary endpoints, there was a significant decrease in maternal insulin use in the probiotics group [58]. Another trial showed that supplementation with *MI*, probiotics, and multiple micronutrients preconception and in pregnancy did not lower gestational glycemia but reduced pre-term birth [36].

Very recently, in agreement with Masulli et al. 2020 [58], Davidson [59] reported no benefits on GDM incidence associated with probiotics. Although no difference in maternal-fetal endpoints has been identified, an increased risk of pre-eclampsia in occurrence with probiotic administration has been observed. This latter data warrants particular consideration, in order to shed light on the underlying potential physiology of the relationship between probiotics and pre-eclampsia.

#### 3.2.2. Probiotics Supplementation and GDM Treatment

Trials on the use of probiotics in the treatment of GDM are scarce. Probiotics may induce beneficial effects on metabolic and neonatal outcomes in GDM women. Taylor et al. [60] observed a significant reduction in insulin resistance (Mean Difference = −0.69; 95% CI −1.24, −0.14, *p* = 0.01) but no significant difference on FPG (Mean Difference = −0.13; 95% CI −0.32, 0.06, *p* = 0.18) or LDL-cholesterol (−0.16; 95% CI −0.45, 0.13, *p* = 0.67) following probiotic supplementation for 6–8 weeks. According to these results, Zheng et al.’s meta-analysis revealed that probiotics supplementation during pregnancy can have beneficial effects on glucose metabolism, but not lipid metabolism, among GDM women [61]. In a double-blind randomized controlled trial, metabolic parameters (fasting plasma glucose (*p* = 0.034), fasting plasma insulin (*p* = 0.001), and HOMA-IR (*p* = 0.001)) showed a significant improvement after four weeks of probiotic supplementation with Bifidobacterium and Lactobacillus in women with GDM in the late-second and early-third trimester [62]. In addition, no difference in weight gain was found between the probiotic group and the placebo group.

In order to evaluate the safety and effectiveness of probiotics in treating women with GDM on maternal and infant outcomes, Okesene-Gafa et al. [63] identified nine RCTs (695 GDM women). A decrease in markers for insulin resistance (HOMA-IR), HOMA-B, and insulin secretion, as well as an increase in quantitative insulin sensitivity check index (QUICKI) was found with probiotics compared with placebo. In addition, there is evidence of a reduction in inflammatory markers (high-sensitivity C-reactive protein (hs-CRP), interleukin 6, and a marker of oxidative stress, malondialdehyde), and an increase in antioxidant total glutathione. With regard to infant outcomes, there is evidence of a reduction in infant hyperbilirubinaemia with probiotics intake compared with a placebo group (RR 0.18, 95% CI 0.05, 0.57). There were no adverse events reported from the trials. As suggested by the same authors, based on the clinical data available, evidence does not support the use of probiotics as a treatment for GDM due to the variability of probiotics used and the small sample sizes of the trials [63].

## 4. Epigenetic Effects of Supplements in Pregnancy

Epigenetic mechanisms, namely DNA methylation, histone modifications, and small non-coding RNAs, can induce heritable changes in gene expression, without a change in DNA sequence. These mechanisms play an important role in a broad range of biological processes at the level of chromatin structure and organization [64]. Although research on in-pregnancy epigenetic modifications which can be transgenerationally inherited has now raised interest in the scientific community, knowledge of their underlying mechanism is still unclear [10,65,66]. The risk of non-communicable chronic conditions in later life can be influenced by epigenetic modifications occurring in utero or during early neonatal stages [64]. Dietary patterns, nutrients, and bioactive compounds have been reported to interact with metabolic traits through epigenetic mechanisms, thus representing a potential and attractive therapeutic target [67].

**Figure 1:**
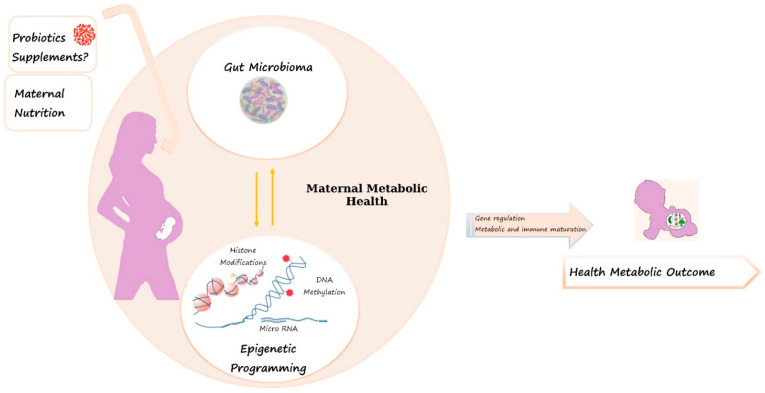
The complex interactions among early nutrition, microbiome, epigenome and long-term health development.

Specific epigenetic modifications after bacteria exposure have been identified, suggesting complex interactions among the microbiome, metabolism, and the epigenome [68] (Figure 1). Vähämiko et al. were the first to study how probiotic supplements modified DNA methylation throughout pregnancy in promoters for obesity-related genes in both mothers and their offspring [69]. In particular, the authors showed that probiotic supplementation during pregnancy may be able to modify the DNA methylation level in the promoter of women’s *FTO* gene, that is, the gene most strongly associated with obesity, body mass index, and T2D in several studies [70,71].

In addition, Vähämiko et al. found hypomethylation both in the promoter for insulin-like growth factor-binding protein-1 (IGFBP1) and for MSRA (methionine sulfoxde reductase A) in both mothers and their offspring from the group that was administered probiotics. Low levels of IGFBP1, which binds insulin-like growth factors I and II, have been associated with insulin resistance and diabetes. These data may partially elucidate the effect of probiotics on glucose metabolism. Future investigations may confirm these observations in primary tissues and in larger populations, as well as with other probiotic strain supplementation.

In addition to epigenetic alterations, some evidence suggests an essential role of GM in mothers’ and children’s metabolism [72]. Some authors suggest that the GM can affect our epigenome. The microbiota may be an additional source of some epigenetic substrates which are co-factors or regulators of chromatin modifications [73]. Furthermore, GM generates folate and B vitamins that donate methyl groups for DNA or histone methylation. In this context, probiotics and prebiotics supplementation before pregnancy can induce an epigenetic effect on the host due to increased B and D vitamins and zinc status, thus improving maternal glycemia and glucose supply to the fetus-placental unit as well as promoting growth and optimal body composition of the offspring [74]. Morovic et al. [75] defined epigenetics as “A New Frontier in Probiotic Research”, distinguishing “epigenetics within an organisms opposed to the influence that an organism may have on the epigenetics of different organisms (‘para-epigenetics’)”. The authors highlighted the importance of assessing epigenetic and para-epigenetic traits of a probiotic organism in order to understand the mechanisms underlying probiotics’ mode of action.

Little is known about the effects of probiotic supplements on the metabolic environment in pregnancy. Probiotic supplements seem to be able to restrain gestational weight gain and blood glucose levels in pregnancy, improving insulin sensitivity, which may have a beneficial impact on the metabolic health in both mothers and their offspring [50,76,77]. Until now, no evidence has confirmed the efficacy of probiotics in the prevention or treatment of GDM. Some research has concluded that microbial gut colonization may begin in utero; therefore, detrimental epigenetic modifications and, consequently, later development of non-communicable chronic conditions, might be prevented by modulating microbial contact in early life [78,79]. Following the so-called “bacteriotherapy” introduced by Patel [80], the modulation of GM through prebiotic, probiotic, and symbiotic supplementation seems a promising approach for rebalancing the homeostasis of systemic immune systems. In this scenario, further research studies are required to gain further insight into this field, aiming both at understanding microbial epigenetic programming during fetal life as well as defining maternal interventions to improve disease prevention.

It is essential to point out that the heterogeneity of the results reported in the relevant literature is due to several factors, among which are the different duration of the treatments, with either single or multiple strains, and the variability of the probiotics used. Preliminary data currently available have identified potential crosslink mechanisms between GM composition, epigenetic regulation, and metabolic disturbance; however, the underlying mechanisms still need to be clearly elucidated.

Further research is warranted to better understand the epigenetic effects of supplementations during pregnancy. Considering the reversibility of epigenetic modifications, identifying specific epigenetic marks may provide an opportunity for future diagnostic, prognostic, and therapeutic approaches in the field of personalized medicine.

## 5. Conclusions

At present, there is an increasing focus on the role that inositol and probiotics supplementation can play in metabolism during pregnancy and in promoting offspring health.

Supplementation with MI 2000 mg + folic acid 200 mcg twice a day has been demonstrated to reduce the incidence of GDM in Caucasic women at risk of this condition. In women with GDM, supplementation with both MI 2000 mg + folic acid 200 mcg twice a day and DCI inositol 500 mg twice a day seems to improve glucose control and some gestational outcomes. In addition, MI 2000 mg + folic acid 200 mcg twice a day can reduce plasma adiponectin and HOMA-IR. Findings on the use of probiotics are more controversial. Some studies conclude that probiotics supplementation does not reduce the incidence of GDM, with a very small, statistically but not clinically significant, reduction in fasting plasma glucose. However, a recent meta-analysis demonstrated the beneficial effects of probiotics supplementation on blood glucose levels, lipid profile, inflammation, microbiome composition, and oxidative markers, which may reduce GDM among pregnant women [81].

In summary, several studies suggested the potential role of these supplements to improve maternal metabolism and pregnancy outcomes [29,30,81,82]. Therefore, at present, nutritional medical therapy and lifestyle intervention are considered the cornerstone for the prevention and treatment of GDM.

Nonetheless, larger studies in populations of different ethnic groups are needed in order to assess the optimal dosage, frequency, and timing of supplementation, as well as the safety and long-term effects on maternal, neonatal, and childhood outcomes.

## Figures and Tables

**Table 1:** Studies on the effect of inositol supplementation to prevent GDM.

Ref.	Study Design	Time to Supplementation	Type of Supplementation	Participants	Main Results
D’Anna et al. 2013 [24]	RCT	From the 12th week of gestation	n = 110 2000 mg MI + 200 μg folic acid twice a day n = 110 200 μg folic acid twice a day	220 Caucasian pregnant women with family history of DM2	Lower incidence of GDM in MI group (*p* = 0.04) Risk decreased by 65% (OR. 0.35) Lower incidence of macrosomia in MI group
Matarrelli et al. 2013 [25]	RCT	From the 12th week of gestation	n = 36 2000 mg MI + 200 μg folic acid twice a day n = 39 200 μg folic acid twice a day	75 women with high fasting glucose in the first trimester	Lower incidence of GDM in MI group (*p* = 0.001) with RR = 0.127 Later delivery in MI group Lower birth weight and abdominal circumference in MI group
D’Anna et al. 2015 [26]	RCT	From the 12th week of gestation	n = 110 2000 mg MI + 200 μg folic acid twice a day n = 110 200 μg folic acid twice a day	220 pregnant obese women	Lower incidence of GDM in MI group (*p* = 0.001; OR = 0.34, 95% CI 0.17–0.68) Reduction in HOMA-IR in MI group (*p* = 0.048)
Santamaria et al. 2016 [27]	RCT	From the 12th week of gestation	n = 110 2000 mg MI + 200 μg folic acid twice a day n = 110 200 μg folic acid twice a day	220 women with pre-pregnancy BMI 25–30 kg/m^2^	Lower incidence of GDM in MI group (*p* = 0.004) (OR 0.33; 95% CI 0.15–0.70)
Godfrey et al. 2021 [36]	RCT	Preconception and during pregnancy	n = 870 Nutritional formulation with MI (4 g/day), probiotics and multiple micronutrients n = 859 Standard micronutrients supplement	1.729 New Zealand women planning conception	No effect on glucose, incidence of GDM or fetal outcomes Lower preterm deliveries in MI group (aRR 0.43 [0.22–0.82]) Lower postpartum hemorrhage in MI group (aRR 0.44 [95% CI 0.20–0.94])
Farren et al. 2017 [32]	RCT	From the 10th week of gestation	n = 120 1100 mg MI + 27.6 mg DCI, 400 μg folic acid n = 120 200 μg folic acid twice a day	240 pregnant women with family history of DM2	The combination MI + DCI does not reduce the incidence of GDM as compared to placebo
Celentano et al. 2020 [4]	RCT	At the first visit in pregnancy	n = 39 2000 mg MI + 200 μg folic acid twice a day n = 32 500 mg DCI + 400 μg folic acid n = 34 1100 mg MI + DCI 27.6 g + 400 μg folic acid n = 52 400 μg folic acid	157 pregnant non-obese women	Lower incidence of GDM in MI group (5.1% versus 61.5% in control group, 34.4% in DCI, and 38.2% in MI/DCI; *p* < 0.001) Lower abdominal circumference and birth weight in MI group

GDM = gestational diabetes mellitus, RCT = randomized controlled trials, RR = risk ratio, aRR = adjusted risk ratio, OR = odds ratio, NNT = number needed to treat, DM2 = diabetes mellitus type 2, MI = myo-inositol, DCI = D-chiro-inositol, BMI = body mass index, HOMA-IR = homeostasis model assessment insulin resistance.

**Table 2:** Studies on inositol supplementation to treat gestational diabetes.

Ref.	Study Design	Time to Supplementation	Type of Supplementation	Participants	Main Results
Corrado et al. 2011 [37]	RCT	From GDM diagnosis	n = 24 2000 mg di MI + 200 mcg folic acid twice a day n = 45 400 mcg folic acid	69 women with GDM	Lower HOMA-IR in MI group (*p* < 0.001) Higher adiponectin in MI group (*p* = 0.009)
Di Biase et al. 2017 [38]	RCT	From GDM diagnosis	n = 67 DCI 500 mg twice a day n = 70 placebo	137 women with GDM	Lower post-prandial glucose (*p* < 0.005), insulin dose (*p* = 0.026), and weight gain (*p* = 0.015) in DCI group Lower abdominal circumference in DCI group (*p* < 0.001)
Fraticelli et al. 2018 [2]	RCT	From GDM diagnosis	n = 20 2000 mg MI + 200 mcg folic acid twice a day n = 20 500 mg DCI + 400 mcg folic acid n = 20 1100 mg MI + 27.6 g DCI + 400 mcg folic acid n = 20 400 mcg folic acid	80 Caucasian women with GDM	Lower HOMA-IR (*p* < 0.001) and weight gain (*p* < 0.005) in MI group Lower need of insulin therapy in MI group Lower insulin dose in MI group Lower birth weight in MI, DCI, and MI/DCI groups (*p* = 0.032)
Pintaudi et al. 2018 [3]	Case-control study	From the 30th week of gestation	n = 6 4000 mg/day MI + 400 mcg folic acid n = 6 400 mcg folic acid	12 Caucasian women with GDM	Lower glycemic variability in MI group (*p* < 0.001) No significant differences on neonatal outcomes
Kulshrestha et al. 2021 [40]	RCT	From GDM diagnosis	n = 50 1000 mg MI twice a day n = 50 control group	100 Asian Indian women with singleton pregnancy and GDM	Lower plasma glucose in MI group (*p* = 0.008) Lower need of insulin treatment in MI group (6.1% vs. 22.0%, *p* = 0.02) Lower birth weight in MI group (*p* = 0.018)

GDM = gestational diabetes mellitus, RCT = randomized controlled trials, MI = myo-inositol, DCI = D-chiro-inositol, BMI = body mass index, HOMA-IR = homeostasis model assessment insulin resistance.
